# The effects of psychological distance on spontaneous justice inferences: A construal level theory perspective

**DOI:** 10.3389/fpsyg.2022.1011497

**Published:** 2022-12-01

**Authors:** Qing Zhang, Wei Wei, Ningchen Li, Wen Cao

**Affiliations:** ^1^College of Politics and Public Administration, Shandong Youth University of Political Science, Jinan, China; ^2^School of Marxism, Chongqing University of Science and Technology, Chongqing, China

**Keywords:** spontaneous justice inferences, morality, psychological distance, construal, spatial and temporal

## Abstract

**Objective:**

The aim of this study was to examine the effects of psychological distance on spontaneous justice inferences.

**Methods:**

Two experiments were conducted using the probe recognition paradigm to directly examine whether psychological distance affects spontaneous justice inferences. In Experiment 1, the spatial distance of justice actions from the perceivers was manipulated. In Experiment 2, temporal distance was manipulated.

**Results:**

Consistent with our expectations, the results of Experiments 1 and 2 (for spatial distance and temporal distance, respectively) consistently demonstrated the effect of psychological distance on spontaneous justice inferences. In concrete terms, participants made stronger spontaneous justice inferences when confronted with situation descriptions of justice-implying events occurring in a spatially distant location than in a proximal location (Experiment 1) and in the distant-future condition than in the near-future condition (Experiment 2).

**Conclusion:**

These findings indicate that psychological distance can influence influence simplicity, unintentional processing of justice inferences.

## Introduction

Although the moral foundations theory argues that there are five different moral foundations, care and justice are two key components of moral character (Lapsley and Lasky, 2001; Aquino and Reed, 2002; Walker and Hennig, 2004). Care reflects the obligation to help and/or protect others and justice reflects respect for moral rules (Haidt and Graham, 2007) (Yudkin et al., 2018; submitted manuscript).^1^ Furthermore, justice is a core issue in almost every social situation (e.g., politics, organizations, and intimate relationships) in which people interact with each other (Folger, 1984). Justice-related actions are a common feature of people’s daily lives. Previous studies about justice have largely focused on explicit justice judgments using explicit measures like Likert-type rating scales (see Lind and Tyler, 1988; Colquitt and Shaw, 2005). A large body of research has shown that perceivers who observe actions often draw inferences spontaneously, called spontaneous social inferences (Kruse and Degner, 2021). Importantly, Research findings have shown that people draw justice inferences spontaneously (unconsciously and/or unintentionally) when they observe a justice-implying event (Ham and van den Bos, 2008, 2011; Ham et al., 2009; Wang et al., 2014). For example, when reading the sentence “Despite getting the same scores, X did not receive praise, while others did,” people have a tendency to spontaneously infer the “unjust” nature of this situation.

To date, evidence for the occurrence of spontaneous justice inferences has been obtained using the probe recognition paradigm (Ham and van den Bos, 2008), which is based on a paradigm developed by McKoon and Ratcliff (1986). In this paradigm, participants are presented with behavioral sentences including both experimental sentences and control sentences. Each sentence is immediately followed by a probe (justice properties), which is implied by the corresponding experimental sentence. Unlike control sentences, experimental sentences imply the probe words, meaning it becomes harder to respond correctly to the experimental sentences. If participants draw the justice properties spontaneously at the encoding of the experimental sentences, they will require a longer period of time to respond to the experimental sentences than the control sentences, and/or will make more errors doing so (i.e., spontaneous justice inferences interfere with task performance). Longer reaction times (RTs) for the experimental sentences suggest that participants were more likely to have made spontaneous justice inferences from the experimental sentences rather than the control sentences. Thus, participants are not inferring justice properties when responding to the probe words; they have already inferred justice properties when presented with the behavioral sentences. RTs in responding to the probe words are evidence that these inferences have been made earlier at exposure (see Uleman et al., 1996; Ham and Vonk, 2003; Wigboldus et al., 2003; Ham and van den Bos, 2008; Yang and Wang, 2016; Orghian et al., 2019).

In the past two decades, the key issue of inevitability has received considerable attention in the field of spontaneous inferences. Recent research suggests that although spontaneous trait inferences are very difficult to prevent, they are not inevitable (Shi et al., 2019). However, are spontaneous justice inferences inevitable when reading about others’ justice-implying behaviors? This may not be the case. Ham and van den Bos (2008) found that personal relevance for justice recipients influenced spontaneous justice inferences. Specifically, spontaneous justice inferences were less strong for justice-implying events happening to somebody else than to themselves (Experiment 1) and to strangers rather than to friends (Experiment 2). The current research aimed to test the possibility that contextual information concerning justice-implying behaviors’ relative psychological distance is crucial in determining how the perceiver interprets these behaviors and that this will affect spontaneous justice inferences.

According to construal level theory (CLT, Liberman and Trope, 2014; Roberts, 2020), the term “psychological distance” is defined as the “subjective experience that something is close or far away from the self, here, and now” (Trope and Liberman, 2010, p. 440). Psychological distance comprises different dimensions, such as the spatial dimension (some other place than here), temporal dimension (the future or past self), social dimension (other or self), and hypotheticality dimension (probable or unlikely). Furthermore, distance is considered to increase the abstractness of information processing. “[T]he […] function of high-level construals is to enable people to mentally transcend the here and now by forming a representation consisting of the invariant features of the available information” (Trope and Liberman, 2010, p. 450). Thus, high (low) psychological distance is related to high (low) mental construal, which refers to the individual’s mindset. High-level construals are mental representations that capture abstract, superordinate (e.g., excelling academically), and decontextualized features. Low-level construals, in contrast, are mental representations that include concrete, subordinate, and contextualized features (e.g., reading a textbook, Eyal and Liberman, 2012; Stegall-Rodriguez and Collette, 2022).

Due to their highly general, schematic, and abstract nature, moral principles constitute high-level constructs with respect to psychologically more distant situations (Hess et al., 2018). As such, although moral behaviors can be interpreted in terms of decontextualized abstract causes and concrete situational factors, considerable evidence has shown that psychologically distant factors (e.g., temporal, spatial, and social) were directly associated with stronger moral judgments with abstract moral causes [see review by Mårtensson (2017)]. For example, studies found that people attribute actors’ temporally distant immoral behavior to more abstract and dispositional moral causes (e.g., he or she was a selfish person), as opposed to concrete and situational causes (Agerström and Björklund, 2009). Moreover, Eyal et al. (2008) found that participants chose transgression of moral principles (e.g., desecrating a national symbol) compared to concrete means (e.g., cutting a flag into rags) to describe temporally distant moral transgression behaviors (e.g., cleaning the house with an old Israeli flag).

However, to our knowledge, the overwhelming majority of CLT-based research on moral judgment focuses on deliberate moral choices (Eyal et al., 2008) and general moral judgment in which participants are merely required to make judgments about whether the moral and immoral actions are good or bad (Li and Rao, 2017). Little attention has been paid to directly exploring the inferring moral properties from moral-implying behaviors. As mentioned before, justice is one of the two key components of moral character and is seen as the core foundation of moral reasoning (Kohlberg, 1969). The application of justice rules is immediate and spontaneous (Haidt, 2001). Moreover, Justice is more closely associated with high-level construals due to its more abstract and decontextualized nature (Agerström et al., 2010). As such, the main aim of the present study was to test the effects of psychological distance on spontaneous justice inferences. Based on previous research on the effect of psychological distance on moral judgment and the above-mentioned considerations, we made the assumption that participants would make stronger spontaneous justice inferences from distant rather than proximal justice-implying behaviors.

As stated before, there are four fundamental psychological distance dimensions. Considerable evidence suggests that some dimensions are more important than others. Due to being learned earlier and their relatively stable and controllable nature, spatial distance may have a more primary nature than other types of psychological distance (Zhang and Wang, 2009; Trope and Liberman, 2010). In addition, temporal distance, which forms the basis of CLT, is by far the most frequently used dimension to investigate the relationship between construal level and moral judgments (Agerström and Björklund, 2009; Agerström et al., 2010; Mårtensson, 2017). Based on the above considerations, two studies were performed to examine the effects of psychological distance on spontaneous justice inferences by manipulating the spatial distance and temporal distance of actions from the perceivers.

In summary, two experiments were conducted using the probe recognition paradigm to directly examine whether psychological distance affects spontaneous justice inferences. In Experiment 1, the spatial distance of justice actions from the perceivers was manipulated. We expected perceivers to form spontaneous justice inferences more stronger for spatially distant actions than spatially near ones. In Experiment 2, temporal distance was manipulated. We expected perceivers to form spontaneous justice inferences more stronger for temporally distant actions than temporally near ones. Importantly, previous research (assessing explicit moral judgments) found effects of psychological distance on moral and justice judgments. We believed that to some extent this study would help understand the relationship between psychological distance and implicit justice and moral judgment.

## Experiment 1

Experiment 1 was designed to test the hypothesis that spatial distance has an effect on spontaneous justice inferences. The experiment examined whether Chinese undergraduates made stronger spontaneous justice inferences from spatially distant justice-implying behaviors compared to the same behaviors that were spatially proximal. In order to manipulate spatial distance, participants were told that behavioral events were either happening approximately 5 kilometers away (proximal condition) or approximately 5,000 kilometers away (distant condition). In order to reduce potential confounds relating to participants’ familiarity and attitude toward actual locations, the names of the locations were not included in this experiment. It was expected that participants would draw more spontaneous justice inferences from justice-implying behaviors happening in the spatially distant condition than in the spatially proximal condition.

### Method

#### Participants and design

A power analysis by G Power (version 3.1.9.7) indicated that 54 participants were required to detect an effect size of 0.25 or higher for the design of this experiment, with a probability of 1–β = 0.95, α = 0.05 (Faul et al., 2007). Taking into account the possibility of invalid or missing data, 70 undergraduates (31 males and 39 females) aged 18 years or above studying at Shandong Youth University of Political Science, P.R. of China, participated in the study for partial course credit. Two experiments were approved by the Institutional Review Board at Shandong Youth University of Political Science. Half of the participants were randomly assigned to the spatially proximal condition and the other half were assigned to the spatially distant condition. The experiment consisted of a 2 (spatial distance: proximal or distant) × 2 (sentence type: experiment or control) mixed ANOVA with the latter factor as the within-subjects variable. Two female participants in the spatially proximal condition had to withdraw from the study because of technical problems.

#### Stimuli

##### Experimental trials

Based on previous studies (e.g., Ham and van den Bos, 2008; Wang et al., 2014), four pairs of justice words (just vs. unjust, fair vs. unfair, justified vs. unjustified, equal vs. unequal) relating to justice in Chinese were selected. In order to create justice-implying behavioral sentences, preliminary research consisting of three stages was performed. In the first stage, 10 undergraduates were asked to write three pairs of behavioral sentences for each pair of justice words. In the second stage, three pairs of justice-implying behavioral sentences for each pair of justice words were selected by the psychology graduates. In the last stage, 30 undergraduates were asked to evaluate each of 12 pairs of behavioral sentences in terms of how well it corresponded to its intended justice words on a 7-point scale (1 = not at all, 7 = extremely). Four pairs of sentences with the highest scores for the corresponding pairs of justice words greater than 5.67 were selected as the final experimental sentences. Although there are many types of justice, such as distributive justice, process justice, interactional justice, and informational justice (Graso et al., 2020), all behavioral sentences in present study were of a distributive justice type. A control version of each pair of experimental sentences was also created, which included the major words of the experimental sentences but did not imply the probe words. The unknown protagonist was denoted by the letter “X” in each of the sentences (see the Table A1 for all the experimental materials).

In order to make sure the control sentence does not imply the probe word but the experimental sentence does, an additional post study was conducted. All of the experimental sentences and the control sentences were presented to 35 college students. They were asked to evaluate on a 7-point scale (1 = not at all, 7 = extremely) to what extent each of these sentences implied the corresponding probe. The results showed that there was a significant difference between the experimental sentences (*M* = 5.53, *SD* = 0.36) and the control sentences (*M* = 2.72, *SD* = 0.54), *t* = 24.23, *p* < 0.001, indicating that the experimental sentences implied justice but the control sentence did not. They were also asked to indicate on a 5-point scale (1 = very easy, 5 = not very easy) how easy it was for them to understand each of the sentences. The results showed that there was no significant difference between experimental sentences (*M* = 1.89, *SD* = 0.23) and control sentences (*M* = 1.80, *SD* = 0.35), *t* = 1.38, *p* = 0.178.

##### Filler trials

If only experimental trials were presented, all of the correct answers were “No.” In order to balance the yes and no answer, we included 36 filler trials developed by Yan and Wang (2011) in addition to the 12 experimental trials.

#### Procedure

Each participant worked individually on a personal computer. At the beginning of the experiment, the participants were told that they were taking part in a memory study. Additionally, half of the participants were told that the events happened approximately 5 kilometers away and the other half were told that the events happened approximately 5,000 kilometers away. Participants were told that they would be shown many different behaviors which would be presented on the screen for 1,000 ms. After a 1,000 ms blank screen, a probe word (in red letters) would appear on the screen after each sentence. Their task was to decide as quickly and accurately as possible whether the word had been seen in the previous sentence. They were asked to press the yes key (the F key, labeled “yes”) if they believed they had seen the word or the no key (the J key, labeled “no”) if they believed they had not seen the word. Each probe word stayed on the screen until the participant’s reaction had been recorded. After each response, there was a blank screen for 800 ms before the next trial started. For each participant, the order of the 48 trials (12 experimental trails and 36 filler trails) was randomized. Before the actual test trials started, the participants were given four practice trials to familiarize them with the task. The computer recorded responses and RTs.

Previous studies indicated that several variables (e.g., familiarity, similarity) vary systematically with spatial distance (Henderson, 2009; Henderson et al., 2011; Soderberg et al., 2015). For example, actions that are distant tend to be less familiar. In order to examine the effects of distance independent of these covariates, three factors were measured after the experiment in order to address potential confounds. For familiarity, participants were asked to rate on a scale (1 = not at all, 7 = extremely) to what extent they were familiar with either the proximal location or the distant location, depending on which condition they were in. Participants were also asked to rate on a scale (1 = not at all, 7 = extremely) to what extent they perceived the targets to be similar to themselves. To measure attitudes, participants were asked to rate how positive they felt toward the target location on a scale (1 = not at all, 7 = extremely).

### Results and discussion

#### Reaction time

Incorrect “Yes” responses to the experimental trials were removed from the RT analysis. There are no standard procedures for dealing with outliers (Uleman et al., 1996); however, in line with the recommendation by Ratcliff (1993) and applied in most studies using the probe recognition paradigm (Wigboldus et al., 2003; Ham and van den Bos, 2008, 2011; Saribay et al., 2012; Yan et al., 2012), RTs faster than 200 ms or slower than 2000 ms were considered as outliers and discarded. As a result, 4.04% had to be dropped from further RT analysis. The effects of participant gender were preliminarily analyzed. Because no effect was found in all of the analyses, this factor will not be discussed further.

The RTs were submitted to a 2 (spatial distance: proximal or distant) × 2 (sentence type: experiment or control) mixed ANOVA. As indicated in Table 1, this analysis yielded a main effect for sentence type, *F*(1,66) = 32.47, *p* < 0.001, partial η2 = 0.33. Participants took more time to correctly recognize probes for the experimental sentences than for the control sentences. This significant main effect was qualified by a significant interaction effect between spatial distance and sentence type, *F*(1,66) = 8.48, *p* < 0.001, partial η2 = 0.11. Further analysis found that in the spatially proximal condition, participants’ RTs for the experimental sentences were longer than for the control sentences, *F*(1,32) = 6.11, *p* = 0.019, partial η2 = 0.16. In the spatially distant condition, RTs for the experimental sentences were also longer than for the control sentences, *F*(1,34) = 28.01, *p* < 0.001, partial η2 = 0.45.

**Table 1:** Mean RTs (in milliseconds) and ERs to the key justice probes as a function of spatial distance and sentence type, with standard deviation in parentheses.

Spatial distance	Sentence type	
	Experimental	Control
RTs (ms)		
Near	897 (220)	839 (194)
Distant	939 (249)	815 (173)
ERs (%)		
Near	4.17 (9.20)	2.27 (7.30)
Distant	3.57 (9.38)	2.86 (8.07)

As mentioned in the introduction, the major assumption of the probe recognition paradigm is that if participants make spontaneous justice inferences from the corresponding behavior when they are exposed to the behavioral sentences, they should exhibit longer RTs for the experimental sentences compared to the control sentences. As such, these results suggested that participants made spontaneous justice inferences in both the spatially distant condition and spatially proximal condition, but the interaction effect between spatial distance and sentence type may be that they made stronger spontaneous justice inferences in the spatially distant condition than in the spatially proximal condition.

To shed further light on the differences in spontaneous justice inference effect between the spatially distant and spatially proximal conditions, a strength tendency score was calculated for each participant by subtracting the RTs for the control sentences from the RTs for experimental sentences (see Newman, 1993; Ham and van den Bos, 2011; Zhang and Fang, 2016; Wang and Yang, 2017). The strength tendency score served as a dependent variable in the independent samples t test. The results showed that the scores for the spatially distant condition (*M* = 124, *SD* = 138) were higher than those for the spatially proximal condition (*M* = 40, *SD* = 93), *t* (66) = 2.91, *p* = 0.005, Cohen’s *d* = 0.71. These results demonstrated that compared to the spatially proximal condition, participants made stronger spontaneous justice inferences in the spatially distant condition.

The effect of spatial distance on spontaneous justice inferences was not explained by differences in similarity, attitude, or familiarity. As expected, participants in the spatially proximal condition and the spatially distant condition did not differ in how similar they perceived themselves to be to the protagonists in the actions and they did not differ in attitude toward the location, *ts* ≤ 1.00, *ps* > 0.05. Although the names of the locations were not included in this experiment, participants in the spatially proximal condition (*M* = 2.58, *SD* = 1.30) were more familiar with the location than those in the spatially distant condition (*M* = 1.85, *SD* = 1.56), *t* (66) = 2.06, *p* < 0.05, Cohen’s *d* = 0.51. However, familiarity was a non-significant covariate, and the main effect for sentence type and the crucial interaction between spatial distance and sentence type remained significant when familiarity was included as a covariate in the main analysis. These results suggested that they do not mediate the effect of spatial distance in spontaneous justice inferences.

#### Error rates

Descriptive statistics for error rates (ERs) are presented in Table 1. Due to the highly skewed distribution of the ERs, a square root transformation was conducted to reduce skew (see Cohen and Cohen, 1975). The resulting data were analyzed in a 2 (spatial distance: proximal or distant) × 2 (sentence type: experiment or control) mixed ANOVA. No effects were found. This is not surprising, considering the simplicity of the task and the low overall ER.

Experiment 1 provided support for the effect of spatial distance on spontaneous justice inferences. In concrete terms, compared to the spatially proximal condition, participants made stronger spontaneous justice inferences in the spatially distant condition.

## Experiment 2

In Experiment 2, our aim was to replicate and provide additional support for the main findings from Experiment 1 by using a different manipulation of psychological distance, time. Previous studies on CLT have shown that when thinking about the future as opposed to the present people use more abstract mental representations and increase the weight of various aspects of morality concerns. Experiment 2 examined how a manipulation of temporal distance affected spontaneous justice inferences. Similar to the findings from Experiment 1, it was hypothesized that compared to participants in near-future condition, participants in the distant-future condition would form more spontaneous justice inferences from the same justice-implying behavioral information.

### Method

#### Participants and design

Sixty-six undergraduates (an equal number of males and females) aged 18 years or above studying at Shandong Youth University of Political Science, P.R. of China, participated in the study. Each of the participants was recruited *via* an invitational WeChat message between November and December 2021 and they signed an informed consent form. Participants were randomly assigned to two between-subjects conditions. Half of the participants were randomly assigned to the near-future condition and the other half were assigned to the distant-future condition. The experiment consisted of a 2 (temporal distance: near or distant future) × 2 (sentence type: experiment or control) mixed ANOVA with the latter factor as the within-subjects variable. One male participant in the near condition had to withdraw from the study because of technical problems.

#### Stimuli

The stimuli were the same as in Experiment 1.

#### Procedure

The procedure was the same as in Experiment 1 except for two differences. As a temporal distance manipulation (Sánchez et al., 2021), half of the participants in the near-future condition were instructed to imagine that the actions would happen in the present (in 2022), while the other half of the participants in the distant-future condition were instructed to imagine that the actions would take place 1 year into the future (in 2023). In addition, similarity was also measured after the experiment in order to exclude alternative explanations. Participants were asked to rate on a scale (1 = not at all, 7 = extremely) to what extent they perceived the targets to be similar to themselves.

### Results and discussion

#### Reaction time

Incorrect “Yes” responses for experimental trials were removed from the RT analysis and RTs faster than 200 ms or slower than 2000 ms were considered as outliers and were discarded, As a result, 2.05% had to be dropped from further RT analysis.

The RTs were submitted to a 2 (temporal distance: near or distant future) × 2 (sentence type: experiment or control) mixed ANOVA. As indicated in Table 2, this analysis yielded a main effect for sentence type, *F*(1,63) = 28.16, *p* < 0.001, partial η2 = 0.31. Participants took more time to correctly recognize probes for the experimental sentences than for the control sentences. This significant main effect was qualified by a significant interaction effect between temporal distance and sentence type, *F*(1,63) = 4.23, *p* = 0.044, partial η2 = 0.06. Further analysis found that in the near-future condition, participants’ RTs for the experimental sentences were longer than for the control sentences, *F*(1,31) = 4.86, *p* = 0.035, partial η2 = 0.14. In the distant-future condition, RTs for the experimental sentences were also longer than for the control sentences, *F*(1,32) = 25.51, *p* < 0.001, partial η2 = 0.48. These results suggested that participants made spontaneous justice inferences in both temporal conditions, but they made stronger spontaneous justice inferences in the distant-future condition than in the near-future condition.

**Table 2:** Mean RTs (in milliseconds) and ERs to the key justice probes as a function of temporal distance and sentence type, with standard deviation in parentheses.

Temporal distance	Sentence type	
	Experimental	Control
RTs (ms)		
Near	824 (138)	778 (145)
Distant	945 (203)	842 (189)
ERs (%)		
Near	4.17 (9.20)	2.27 (7.30)
Distant	3.57 (9.38)	2.86 (8.07)

As in Experiment 1, a strength tendency score was calculated for each participant by subtracting the RTs for the control sentences from the RTs for the experimental sentences. The strength tendency score served as a dependent variable in the independent samples t test. The results showed that the scores for the distant-future condition (*M* = 103, *SD* = 109) were higher than those for the near-future condition (*M* = 46, *SD* = 117), *t* (63) = 2.06, *p* = 0.044, Cohen’s *d* = 0.50. These results demonstrated that compared to the near-future condition, participants made stronger spontaneous justice inferences in the distant-future condition.

The effect of temporal distance on spontaneous justice inferences, however, was not explained by differences in similarity. As expected, participants in the near-future condition (*M* = 3.63, *SD* = 0.98) and participants in the distant-future condition (*M* = 3.58, *SD* = 1.17) did not differ in their perceived similarity to the targets, *t* (63) = 0.18, *p* > 0.05.

#### Error rates

For the ERs, no effects were found. Descriptive statistics for ERs are presented in Table 2.

Overall, Experiment 2 provided evidence on the effect of temporal distance on spontaneous justice inferences. In concrete terms, compared to the near-future condition, participants made stronger spontaneous justice inferences in the distant-future condition.

## General discussion

To our knowledge, the present research provides the first evidence of the effect of psychological distance on the social-cognitive processes of spontaneous justice inferences. In the current research, using a probe recognition paradigm, two experiments were conducted to examine the effects of psychological distance on spontaneous justice inferences. Consistent with our expectations, the results of Experiments 1 and 2 (for spatial distance and temporal distance, respectively) consistently demonstrated the effect of psychological distance on spontaneous justice inferences. In concrete terms, participants made stronger spontaneous justice inferences when confronted with situation descriptions of justice-implying events occurring in a spatially distant location than in a proximal location (Experiment 1) and in the distant-future condition than in the near-future condition (Experiment 2). The results of present study consisted with the prior studies which have found that psychologically distant (e.g., temporal, spatial, and social) were directly associated with stronger moral judgments with abstract moral principles [see review by Martensson (2017)].

These findings are fully in line with predictions derived from CLT (see Eyal et al., 2008). Based on the view of CLT, a feeling of greater psychological distance from events induces individuals to engage in abstract thinking, thus leading them to be more likely to automatically activate justice principles which constitute the general abstract concept of morality (Eyal and Liberman, 2012). The accessibility of justice principles, in turn, might be more likely to guide people’s subsequent justice judgments. It is possible that by providing additional psychological distance information, the activated justice principles lower the threshold for justice inferences from justice-implying behavior. As such, the spontaneous justice inferences from psychologically distant actions may be facilitated. However, an important problem remains unresolved: Is the influence of psychological distance on spontaneous justice inferences driven by giving additional distance information to enhance a person’s ability to extract the justice meanings and/or giving additional proximity information to inhibit a person’s ability to extract the justice meanings? Because of the lack of a control condition, the present study does not provide a definitive answer to this question. Some researchers have argued that when lacking distance information, perceivers may feel relatively distant from targets (Rim et al., 2009). Nevertheless, more research is needed to address this issue.

Since the four dimensions of psychological distance operate using similar cognitive mechanisms and have similar effects on perception, evaluations, and action (Trope and Liberman, 2010; Adelina and Feldman, 2021), similar effects on spontaneous justice inferences should be obtained with other dimensions, such as social distance or hypotheticality. However, Ham and van den Bos (2008) found that personal relevance influenced spontaneous justice inferences. These results indicated that a closer social distance (self vs. other; friends vs. stranger) resulted in less strong spontaneous justice inferences. These seemingly contradictory results may be due to social distance manipulation. According to CLT, the behavior of opinions of socially distant people is construed as more abstract than one’s own behavior (Trope et al., 2007; Trope and Liberman, 2010). That is, when social distance was manipulated by asking participants to imagine moral events from the perspective of themselves or from the perspective of another person (Broemer et al., 2008; Eyal et al., 2008, Study 3; Žeželj and Jokić, 2014), moral judgments were stronger with increased social distance. By contrast, when social distance was manipulated by the kinds of justice recipients’ specific features such as group membership, moral judgments were stronger with decreased social distance. Previous studies showed that participants tended to grant greater justice protection to close recipients in comparison with strangers (see Wenzel, 2001; Hafer et al., 2012). Relatedly, perceived interpersonal similarity leads to greater moral inclusion (Hafer and Olson, 2003). Furthermore, Mentovich et al. (2016), study 1 showed that participants were more willing to grant freedom of speech privileges to targets closer to them (such as themselves or their family) compared with remote targets (such as noncitizens and terrorists). Considering the social distance manipulation in Ham and van den Bos’ study for justice recipients, their results are not surprising. Admittedly, this account is speculative and should be examined empirically in future research.

Another issue that deserves comment is that the results of the present study are in apparent contradiction to the moral parochialism reported in Fessler et al. (2015), which found that moral condemnation was more severe when transpiring in the here and now than when occurring at a distance due to convergent cost/benefit incentives from evolutionary functional approaches. Without further study, interpretation of these differences can only be speculative. Fessler et al. (2016) study focused on the moral condemnation of moral transgressions, while The present study examined the implicit attribution process for justice-related behavior. The benefits of moral condemnation come mainly from reputation enhancement and the cost/benefit ratio of harmful behaviors (Fessler et al., 2016). The implicit attribution process, however, is concerned with how to explain behavior. Although it’s not clear whether there are cognitive mechanisms that differ between the two, studies found that there are differences in some ways. For example, recent study (Holbrook et al., 2022) found that although Korean and American participants showed similar moral parochialism in moral condemnation, Korean participants were more likely than American ones to attribute transgressive behavior to situational and contextual factors. Moreover, Taniguchi and Ikegami (2021) found that participants were more likely to apply implicit attribution to a victim’ s internal disposition (carelessness) from an accident that occurred at a distant relative to the near location, which is consistent with the CLT theory. These evidence suggested that implicit attributions (such as spontaneous justice inferences) and explicit moral condemnation might be two different processes. In addition, another difference between the two studies was that Fessler et al. (2015) focused on condemnation of actors, whereas our experiments focused on justice recipients. Studies have found that psychological distance helped participants to attenuate the influence of the idiosyncratic characteristics of justice recipients (such as in-group members and close others) and to increase justice judgments more in line with universalism rather than parochialism (Mentovich et al., 2016). This difference might also account for the different results.

The present research expands on previous CLT research in at least two ways. First, previous research on CLT and moral judgment has mainly focused on hypothetical moral dilemmas (Eyal et al., 2008). The present study showed that the effects of psychological distance fruitfully extended to implicit unintentional processing of justice inferences. Second, the present study also extends the literature on CLT and spontaneous social inference. Previous research has found that psychological distance affects spontaneous trait inferences (STIs, Rim et al., 2009; Taniguchi and Ikegami, 2021). It should be noted that STIs reflect spontaneously dispositional attribution processes made about the actor (Trait A is a property of actor X). For example, if people read the sentence “Although two students got the same score, X praised one but not the other,” people may spontaneously infer that X is “unfair.” In contrast, spontaneous justice inferences measured in the present study are not made about actors but about recipients or actions (see the Table A1). The present study, in this sense, was not merely designed to apply the conclusions from the effect of psychological distance on STIs to the justice domain.

The findings of the present study also foster our understanding of the inevitability of spontaneous justice inferences. Existing research (Brickman et al., 1981) has shown that justice judgments are influenced by both micro-level concerns (target-specific features) and macro-level concerns (relevant justice principles). Ham and van den Bos (2008) found that target-specific features affect spontaneous justice inferences from justice-implying events. The present study further demonstrated that psychological distance which actives relevant justice principles also influenced spontaneous justice inferences. The results of the present study together with those of Ham and van den Bos (2008) clearly demonstrate that although occurring spontaneously, spontaneous justice inferences are apparently not inevitable. They could also be influenced by both macro- and micro-level concerns. Indeed, increasing the psychological distance from justice judgments reduces the weight assigned to specific features of targets (Mentovich et al., 2016). Further studies examining the interaction between psychological distance and target-specific features on spontaneous justice inferences are needed.

In addition, two limitations are worth noting. One limitation is that the present study focused only on distributive justice. Studies have found that the dimension of justice consists of three other components: procedural justice, interactional justice and informational justice. Cojuharenco et al. (2011) found that interactional justice concerns are more salient at lower levels of construal, whereas distributive justice concerns are more salient at higher levels of construal. Future studies examining the impact of the other components of overall justice may further clarify the effects of psychological distance on spontaneous justice inferences.

Another limitation is that this study manipulated construal levels indirectly through the induction of psychological distance. Although a considerable number of studies have confirmed the effects of psychological distance on abstraction (Soderberg et al., 2015; Sánchez et al., 2021), a few recent studies did not find a predicted effect of psychological distance on abstraction (see Maier et al., 2022). Future research could expand this study by directly manipulating the level of construal by either having participants adopt an abstract or a specific mindset (see Fujita et al., 2006, Study 3; Rim et al., 2009).

## Data availability statement

The raw data supporting the conclusions of this article will be made available by the authors, without undue reservation.

## Ethics statement

The studies involving human participants were reviewed and approved by the Institutional Review Board at Shandong Youth University of Political Science. The patients/participants provided their written informed consent to participate in this study.

## Author contributions

QZ designed the experiments and carried out the experiments. WW and WC analyzed experimental results and wrote the manuscript. NL revised manuscript critically for important intellectual content and collected additional data. All authors contributed to the article and approved the submitted version.

## Funding

This research was supported by the Shandong Social Science Planning Fund Program (Project No. 19CXSXJ38).

## Conflict of interest

The authors declare that the research was conducted in the absence of any commercial or financial relationships that could be construed as a potential conflict of interest.

## Publisher’s note

All claims expressed in this article are solely those of the authors and do not necessarily represent those of their affiliated organizations, or those of the publisher, the editors and the reviewers. Any product that may be evaluated in this article, or claim that may be made by its manufacturer, is not guaranteed or endorsed by the publisher.

## Footnotes

^1^Yudkin, D., Mentovich, A., and Trope, Y. (2018). Justice across time. Manuscript submitted for publication. doi: 10.31234/osf.io/pjmv2

## Appendix

**Table A1:** Experimental sentences and the subsequent probes used for the experimental trials in Experiment 1 and 2.

Sentence	Probe
Just implying	
Doing the same job, X makes 2000 yuan, and other people make 2000 yuan.	Equal
For the same score, X was praised by the teacher, and the other person was praised by the teacher	Just
With the same score ranking, X was admitted by the company, and others were admitted by the company	Fair
To participate in the sports meeting, the teacher prepared sports clothes for X, and did so for others	Justified
Unjust implying	
Doing the same job, X makes 2000 yuan, but other people make 4,000 yuan.	Unequal
For the same score, X was not praised by the teacher, but the other person was praised by the teacher	Unjust
With the same score ranking, X was not admitted by the company, while others were admitted by the company	Unfair
To participate in the sports meeting, the teacher did not prepare sports clothes for X, but did so for others	Unjustified
Control	
Doing the same job, X spent 20 h, but other people spent 40 h	Unequal
For the same score, X feels happy and others feel happy	Just
With the same score ranking, X feel excited, and the others feel excited	Fair
To participate in the sports meeting, X’ mother prepared sportswear for X	Unjustified
